# Aripiprazole and Brexpiprazole as Adjunctive Therapies for Difficult-to-Treat Depression: A Literature Review of Clinical Effectiveness, Safety, and Cost-Effectiveness

**DOI:** 10.1192/j.eurpsy.2026.11423

**Published:** 2026-07-31

**Authors:** D. N. Nguyen, A. Soubolsky, H. Lafoy, A. Wanson, K. Halpape

**Affiliations:** 1 College of Pharmacy and Nutrition, University of Saskatchewan; 2 Pharmacy; 3 Psychiatry, Saskatchewan Health Authority, Saskatoon, Canada

## Abstract

**Introduction:**

Difficult-to-treat depression (DTD) affects approximately 30% of individuals with major depressive disorder (MDD), representing a significant clinical and economic burden. Partial dopamine agonist antipsychotics, including aripiprazole and brexpiprazole, are recommended by recent clinical guidelines as first-line adjunctive options for DTD. However, coverage and access remain inconsistent across jurisdictions.

**Objectives:**

This literature review sought to identify, evaluate, and summarize evidence on the clinical effectiveness, safety, cost-effectiveness, and guideline recommendations regarding aripiprazole and brexpiprazole as adjunctive treatments for adults with MDD.

**Methods:**

A systematic search of MEDLINE, Embase, and PsycINFO was conducted from inception to July 2025. Eligible publications included randomized controlled trials, observational studies, pharmacoeconomic evaluations, and evidence-based guidelines published in English. Studies were screened by two reviewers and appraised for validity and clinical relevance.

**Results:**

A total of 21 aripiprazole studies and 17 brexpiprazole studies were included, along with eight cost-effectiveness analyses and 7 clinical practice guidelines. Across these trials, 81% of aripiprazole studies and 88% of brexpiprazole studies demonstrated significant benefit, with mean reductions in Montgomery-Åsberg Depression Rating Scale scores ranging from -4.6 to -19.6. Common adverse effects included akathisia, weight gain, insomnia, and restlessness, with discontinuation due to adverse events occurring more frequently than with placebo. Most cost-effectiveness analyses found both medications to be more cost-effective than other adjunctive options, particularly when initiated earlier. Guideline recommendations were mixed: the 2023 Canadian Network for Mood and Anxiety Treatments (CANMAT) guidelines endorse both agents as first-line adjuncts for DTD, whereas some of the other guidelines list them as adjunctive options without consistent first-line endorsement.

**Conclusions:**

Aripiprazole and brexpiprazole represent evidence-based atypical antipsychotic options for adjunctive treatment of DTD. While effect sizes are modest and adverse effects clinically relevant, untreated depression carries profound personal and societal costs. Expanding formulary access to these medications would align health system coverage with contemporary guideline recommendations, reduce inequities, and improve outcomes in patients with DTD.

**Disclosure of Interest:**

None Declared

